# Associations between the Duration of Dialysis, Endotoxemia, Monocyte Chemoattractant Protein-1, and the Effects of a Short-Dwell Exchange in Patients Requiring Continuous Ambulatory Peritoneal Dialysis

**DOI:** 10.1371/journal.pone.0109558

**Published:** 2014-10-06

**Authors:** Chia-Lin Wu, Hung-Ming Wu, Ping-Fang Chiu, Hung-Hsiang Liou, Chirn-Bin Chang, Der-Cherng Tarng, Chia-Chu Chang

**Affiliations:** 1 Division of Nephrology, Department of Internal Medicine, Changhua Christian Hospital, Changhua, Taiwan; 2 School of Medicine, Chung-Shan Medical University, Taichung, Taiwan; 3 Institute of Clinical Medicine, National Yang-Ming University, Taipei, Taiwan; 4 Inflammation Research and Drug Development Center, Changhua Christian Hospital, Changhua, Taiwan; 5 Division of Nephrology, Department of Internal Medicine, Hsin Jen Hospital, Taipei, Taiwan; 6 Division of Nephrology, Department of Medicine, Taipei Veterans General Hospital, Taipei, Taiwan; 7 Department and Institute of Physiology, National Yang-Ming University, Taipei, Taiwan; University of Leicester, United Kingdom

## Abstract

**Background:**

Endotoxemia is exaggerated and contributes to systemic inflammation and atherosclerosis in patients requiring continuous ambulatory peritoneal dialysis (CAPD). The risk of mortality is substantially increased in patients requiring CAPD for >2 years. However, little is known about the effects of long-term CAPD on circulating endotoxin and cytokine levels. Therefore, the present study evaluated the associations between plasma endotoxin levels, cytokine levels, and clinical parameters with the effects of a short-dwell exchange on endotoxemia and cytokine levels in patients on long-term CAPD.

**Methods:**

A total of 26 patients were enrolled and divided into two groups (short-term or long-term CAPD) according to the 2-year duration of CAPD. Plasma endotoxin and cytokine levels were measured before and after a short-dwell exchange (4-h dwell) during a peritoneal equilibration test (a standardized method to evaluate the solute transport function of peritoneal membrane). These data were analyzed to determine the relationship of circulating endotoxemia, cytokines and clinical characteristics between the two groups.

**Results:**

Plasma endotoxin and monocyte chemotactic protein-1 (MCP-1) levels were significantly elevated in the long-term group. PD duration was significantly correlated with plasma endotoxin (*r* = 0.479, *P* = 0.016) and MCP-1 (*r* = 0.486, *P* = 0.012). PD duration was also independently associated with plasma MCP-1 levels in multivariate regression. Plasma MCP-1 levels tended to decrease (13.3% reduction, *P* = 0.077) though endotoxin levels did not decrease in the long-term PD group after the 4-h short-dwell exchange.

**Conclusion:**

Long-term PD may result in exaggerated endotoxemia and elevated plasma MCP-1 levels. The duration of PD was significantly correlated with circulating endotoxin and MCP-1 levels, and was an independent predictor of plasma MCP-1 levels. Short-dwell exchange seemed to have favorable effects on circulating MCP-1 levels in patients on long-term PD.

## Introduction

Peritoneal dialysis (PD) is a well-established treatment modality for patients with end-stage renal disease. In Taiwan, about 6000 (10% of all patients undergoing dialysis) patients received PD in 2009 (Taiwan Renal Registry Data System, 2009) [1], [2]. Patients treated with PD may have better independence, mobility, and quality of life than patients treated with hemodialysis (HD) [3]. Prior studies also revealed that PD confers a survival advantage compared with HD during the first 2 years of dialysis [4], [5]. However, beyond 2 years, PD was associated with greater risk of mortality compared with HD [4]. Indeed, the long-term survival rate of patients undergoing PD is still poor (about 42% at 5 years) despite continued developments in modern medicine [6].

Cardiovascular disease, infection, and peritoneal fibrosis are common and clinically important complications of PD. Cardiovascular diseases are the most common causes of death in dialysis patients. Cardiovascular risk factors in PD patients include traditional (age, male gender, smoking, hypertension, diabetes and dyslipidemia), non-traditional (anemia, calcium-phosphate complex, inflammation, oxidative stress, etc.), and dialysis-related (bacteremia, hyperglycemia, and fluid overload) risk factors [7], [8].

Endotoxin, also known as a bacterial lipopolysaccharide (LPS), is expressed on the outer membrane of the cell wall of Gram-negative bacterial pathogens, especially *Escherichia coli* [9], [10]. Circulating endotoxin levels are higher in patients with advanced chronic kidney disease (CKD) and in dialysis patients than in other groups of patients [10]. Endotoxemia is also indicative of systemic inflammation, accelerated atherosclerosis, and malnutrition, and contributes to high rates of cardiovascular disease and mortality in dialysis patients [10]–[12]. Endotoxin was considered to be a modifiable factor for inflammation and nutrition in HD patients [13], [14], although no interventional studies for reducing endotoxin levels in PD patients have been performed.

Proinflammatory cytokines, such as interleukin-6 (IL-6) and tumor necrosis factor (TNF), are elevated and are associated with hypoalbuminemia, left ventricular diastolic dysfunction, and mortality in PD patients [15]–[18]. Some studies have also demonstrated that the levels of anti-inflammatory cytokines, such as IL-10 and IL-1 receptor antagonist (IL-1RA), are elevated in PD patients [19], [20].

The dwell time (the period of the dialysis solution in abdomen) has an important impact on dialysis adequacy (i.e., Kt/V_urea_) and ultrafiltration. Long-dwell exchanges may enhance the clearance of uremic toxins such as creatinine and phosphate. However, a prolonged dwell time with a glucose-based solution actually decreases ultrafiltration because of the progressive reduction of the osmotic gradient. In a recent study, a longer daily dwell time was associated with peritoneal epithelial-to-mesenchymal transition [21]. Short-dwell exchanges (dwell time below 4 hours) may provide greater ultrafiltration, and improvements in blood pressure and fluid control, especially in patients with ultrafiltration failure [22]. However, it is unclear whether short- or long-dwell time has better effects on reducing peritoneal fibrosis, inflammation, and atherosclerosis.

To our knowledge, there is little information regarding the relationship between long-term PD, circulating endotoxin levels, and pro- and anti-inflammatory cytokine levels. Furthermore, no studies have demonstrated whether short-dwell dialysis reduces endotoxemia or inflammatory mediators. We hypothesized that endotoxin and proinflammatory cytokines may accumulate in patients requiring long-term CAPD and that their levels would be attenuated by a shorter dwell time compared with a longer dwell time.

## Materials and Methods

### Patients

Between October 1, 2011 and July 31, 2012, we performed an interventional, non-randomised, single-arm, pilot clinical study of 26 stable patients on CAPD at Changhua Christian Hospital PD center. The study was approved by the Institutional Review Board of Changhua Christian Hospital (approval number: 110317). All of the patients were treated with usual dwell time (4–6 hours during the day and 8–10 hours at night), clinically stable and without evidence of an active infection, and all received conventional glucose-based dialysates (Dianeal; Baxter Healthcare SA, Singapore Branch, Singapore). None of the patients were being treated with non-glucose-based PD solutions (e.g., icodextrin), immunosuppressants, non-steroidal anti-inflammatory drugs, iron supplements, or antioxidants such as vitamins C or E. All of the participants provided written informed consent before two blood tests to measure endotoxin and cytokine levels on the day of a peritoneal equilibration test (PET). All patients visited the clinic after an overnight fast and underwent conventional blood tests as well as additional tests to measure circulating endotoxin and cytokine levels.

### Blood and urine sampling

Complete blood cell count, blood urea nitrogen, urinary urea nitrogen, serum creatinine, dialysate creatinine, urinary creatinine, serum high-sensitivity C-reactive protein (hs-CRP), total cholesterol, triglyceride, low-density lipoprotein cholesterol, high-density lipoprotein cholesterol, serum glucose, dialysate glucose, glycated hemoglobin, serum albumin, ferritin, iron, transferrin, and intact parathormone levels were measured using standardized procedures at the Department of Laboratory Medicine at Changhua Christian Hospital.

### PET

Peritoneal equilibration test (PET) is a well-established method to determine the small solute transport function of peritoneal membrane [23]. Higher peritoneal solute transport rate has been linked to worse survival in PD patients [15]. We used a standardized procedure for PET, as proposed by Twardowski et al. [23]. In brief, the collection of peritoneal dialysate samples at several time intervals (0 hour, 2 hours and 4 hours) and a mid-point (2 hours) blood sample for measurement of urea, glucose and creatinine were obtained during the 4-hour exchange with 2 liter of 2.5% glucose dialysate. Dialysate to plasma ratios (D/P) at 4 hours for creatinine were used to quantify and classify individual peritoneal membrane characteristics.

### Circulating endotoxin and cytokine levels

Blood samples for plasma endotoxin and various cytokines were obtained twice on the day of the PET. The first sample was collected after a long overnight dwell and the second sample was obtained after a subsequent short, 4-h dwell. Serum endotoxin levels were measured by a chromogenic Limulus Amebocyte Lysate assay (QCL-1000; Lonza, Walkersville, MD) as previously described [24]. The lower limit of detection was 0.1 endotoxin units (EU)/mL. Plasma levels of the proinflammatory cytokines IL-1β, IL-6, IL-8, IL-12p70, granulocyte-macrophage colony-stimulating factor (GM-CSF), interferon-γ (IFN-γ), and TNF were measured using a highly sensitive magnetic bead-based assay kit (MILLIPLEX MAP Kit, High Sensitivity Human Cytokine Magnetic Bead Kit – Premixed; EMD Millipore, Billerica, MA, USA) [25]. The levels of the proinflammatory cytokine monocyte chemoattractant protein-1 (MCP-1) and the anti-inflammatory mediators IL-10 and IL-1 receptor antagonist (IL-1RA) were assessed using another magnetic bead-based kit (MILLIPLEX MAP Kit, Human Cytokine/Chemokine Magnetic Bead Panel; EMD Millipore) [26]. All assays were performed in accordance with the manufacturers' instructions.

### Statistical analysis

Results are expressed as the percentage, median (interquartile range) or mean ± standard error of the mean. Each variable was tested for normal distribution using the Shapiro-Wilk test. Variables were analyzed using nonparametric statistical tests. Comparisons between two groups were made using the Mann–Whitney *U* test for continuous variables or the χ^2^ test for categorical variables. The Wilcoxon signed-rank test was used to compare paired data. Correlations between pairs of continuous variables were determined by the Spearman rank correlation test. The multivariate regression analysis was performed to predict the independent influence of clinical/biological parameters on duration of PD. The significant variables in correlation analysis (age, Charlson's comorbidity index) and gender, diabetes, serum albumin, hs-CRP, plasma IL-1b, IL-6 and duration of PD were forced into the model to predict circulating endotoxin and MCP-1. All statistical analyses (including principal component analysis) were performed using SPSS Statistics for Windows, version 15 (SPSS Inc., Chicago, IL, USA). In all analyses, *P*-values of <0.05 were considered statistically significant.

## Results

### Patient characteristics

Twenty-six patients (17 men, 9 women) at our PD center were enrolled in this study and their data were suitable for statistical analysis. The mean age was 46.4 years (range, 21.1–62.7 years) and the median time on PD was 23.6 months (range, 1.1–134.4 months). Table 1 shows the baseline demographic and clinical characteristics of these patients. A principal-component analysis (PCA) was performed to reveal the major sources of variation of demographic, clinical and laboratory profiles in the dataset (Figure S1).

**Table 1:** Demographics and clinical characteristics of the study patients.

Variable	Value
n	26
Sex (men/women)	17/9
Age (years)	46.4±1.9
Time on PD (months)	23.6 (7.1–67.7)
Primary renal diagnosis, n (%)	
Glomerulonephritis	13 (50)
Diabetic nephropathy	3 (11.5)
Hypertension	2 (7.7)
Tubulointerstitial disease	2 (7.7)
Polycystic kidney disease	2 (7.7)
Unknown	4 (15.4)
Comorbidities, n (%)	
Diabetes	8 (30.8)
Cardiovascular disease	8 (30.8)
CCI	2.5 (2–4)
BSA (/m^2^)	1.76 (1.51–1.85)
BMI (kg/m^2^)	23.6±0.7
WBC (/µL)	6550 (4850–8950)
Hemoglobin (g/dL)	9.9±0.3
Albumin (g/dL)	3.1±0.1
HbA1c (%)	5.6±0.2
Cholesterol (mg/dL)	182.7±8.0
Triglyceride (mg/dL)	92 (67–128.3)
LDL-C (mg/dL)	150.8±12.5
HDL-C (mg/dL)	48.8±4.4
Uric acid (mg/dL)	6.0±0.3
BUN (mg/dL)	61.2±3.3
Serum creatinine (mg/dL)	12.0±0.7
Serum Ca (mg/dL)	9.4±0.2
Serum P (mg/dL)	5.7±0.3
iPTH (pg/mL)	367.7 (182.8–666.1)
4-h D/P creatinine	0.67±0.028

Values are median (interquartile range), means±standard error of the mean or n (%).

4-h D/P creatinine, dialysate/plasma creatinine ratio at 4 hours; BSA, body surface area; BMI, body mass index; BUN, blood urea nitrogen; CCI, Charlson's comorbidity index; Ca, calcium; HbA1c, glycated hemoglobin; HDL-C, high-density lipoprotein cholesterol; iPTH, intact parathyroid hormone; LDL-C, low-density lipoprotein cholesterol; P, phosphorus; PD, peritoneal dialysis; WBC, white blood cell count.

### Comparisons between the short-term and long-term PD groups

Patients whose duration of PD was greater than 2 years (long-term PD group) were younger, had a lower Charlson's comorbidity index (CCI), and higher circulating intact parathyroid hormone (iPTH), endotoxin and MCP-1 levels than patients with short-term PD. Table 2 compares the demographic, laboratory, and dialysis-related data, as well as circulating endotoxin and cytokine levels between the short-term and long-term PD groups.

**Table 2:** Comparison of demographic and laboratory data between the short- and long-term PD groups.

	Short-term PD (n = 14)		Long-term PD (n = 12)		*P*-value
	Average±SD	Range	Average±SD	Range	
Demographic data					
Age (years)	49.4±10.8	21.1–62.7	43.0±7.2	28–57	0.02
Male gender (%)	78.6		50.0		0.22
BMI (kg/m^2^)	24.3±3.7	16.1–30.3	22.8±3.8	17.4–29.4	0.20
SBP (mmHg)	133.1±21.1	80–150	139.7±12.7	120–150	0.54
DBP (mmHg)	81.3±12.1	60–100	80.1±9.9	65–96	0.69
CCI	3.43±1.40	2–6	2.42±0.67	2–4	0.049
Diabetes, n (%)	6 (42.9)		2 (16.7)		0.22
History of CAD, n (%)	5 (35.7)		3 (25.0)		0.68
PD duration (months)	10.9±8.6	1.1–23.7	72.8±34.0	32.6–134.4	<0.01
Laboratory data					
WBC (/µL)	8278.6±5139.3	3200–23900	6433.3±3575.3	600–15100	0.34
Hemoglobin (g/dL)	9.7±1.9	6.4–12.2	10.0±1.3	7.1–11.6	0.86
Albumin (g/dL)	3.1±0.4	2.3–3.7	3.3±0.3	2.9–3.7	0.25
hs-CRP (mg/dL)	1.05±1.71	0.01–5.90	0.77±1.73	0.01–6.18	0.51
HbA1c (%)	5.8±1.0	3.7–7.9	5.3±0.4	4.9–6.0	0.09
Creatinine (mg/dL)	11.9±4.0	7.0–19.3	12.0±2.9	6.6–15.7	0.82
Ferritin (ng/mL)	284.3±311.4	16.6–1188.6	308.8±376.0	13.7–1331.0	0.88
TSAT (%)	25.5±10.3	11.0–49.7	30.0±14.0	3.4–52.9	0.24
Dialysis-related variables					
4-h D/P creatinine	0.72±0.14	0.53–0.93	0.61±0.13	0.40–0.82	0.05
Weekly CrCL (L/week)	66.6±15.4	50.7–94.1	61.9±12.9	44.0–94.7	0.64
iPTH (pg/mL)	320.9±275.7	26.0–907.3	580.7±272.3	277.0–1324.4	0.02
nPNA (g/kg/day)	0.96±0.28	0.66–1.64	1.00±0.18	0.79–1.49	0.44
Endotoxin (EU/mL)	9.5±15.4	0–58.8	22.6±24.6	2.5–82.2	0.02
Cytokines					
MCP-1 (pg/mL)	301.0±111.5	159–563	489.4±263.7	270–1004	0.04
IL-1β (pg/mL)	4.3±4.0	0–11.9	4.6±5.2	0–16.4	0.92
IL-1RA (pg/mL)	129.3±53.6	42.3–236.0	157.4±102.4	4.8–390.0	0.46
IL-1β/IL-1RA ratio	0.030±0.026	0–0.066	0.027±0.027	0–0.068	0.88
IL-6 (pg/mL)	18.4±13.2	1.9–51.2	15.2±13.9	1.6–53.7	0.22
IL-8 (pg/mL)	21.6±6.7	7.5–31.5	23.5±8.4	12.6–46.0	0.96
IL-10 (pg/mL)	52.0±38.1	9.0–151.0	59.7±70.1	3.0–256.0	0.84
IL-12p70 (pg/mL)	16.39±13.13	0.01–42.38	15.29±14.57	0.01–40.37	0.80
TNF (pg/mL)	28.8±10.2	14.2–49.4	30.6±10.6	16.5–53.5	0.72
GM-CSF (pg/mL)	14.70±12.20	0.01–39.28	14.72±15.30	0.01–43.34	0.88
IFN-γ (pg/mL)	35.8±29.2	5.2–103.0	35.0±33.0	1.4–93.5	0.84

Values are means±standard deviation (SD), range or n (%).

CAD, coronary artery disease; CrCL, creatinine clearance; DBP, diastolic blood pressure; GM-CSF, granulocyte-macrophage colony-stimulating factor; hs-CRP, high-sensitivity C-reactive protein; IFN-γ, interferon-γ; IL, interleukin; IL-1RA, interleukin-1 receptor antagonist; MCP-1, monocyte chemoattractant protein-1; nPNA, normalized protein nitrogen appearance; SBP, systolic blood pressure; TNF, tumor necrosis factor; TSAT, transferrin saturation.

### Correlations between patient characteristics and blood parameters

**Figure 1:**
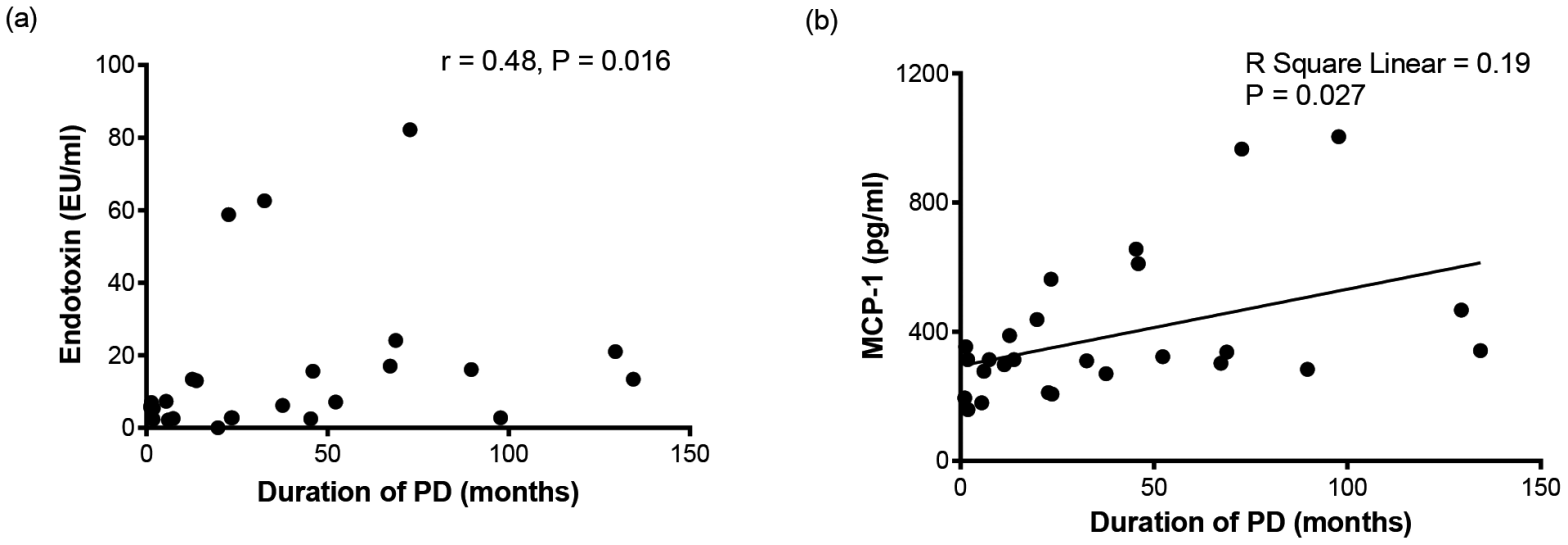
Correlations between the duration of peritoneal dialysis and serum endotoxin (a) and serum MCP-1 (b). A positive association between the duration of PD and serum endotoxin (a) and linear association between the duration of PD and serum monocyte chemoattractant protein-1 (b) levels in patients on CAPD.

Age (49.4±2.9 years in short-term PD and 43.0±2.1 years in long-term PD, *P* = 0.02), CCI (3 (2–5) in short-term PD and 2 (2–3) in long-term PD, *P* = 0.049), and circulating iPTH (246.1 (132.9–447.3) pg/ml in short-term PD and 537 (397.7–684.8) pg/ml in long-term PD, *P* = 0.02), endotoxin (5.4 (2.5–10.2) EU/ml in short-term PD and 15.9 (6.4–23.3) EU/ml in long-term PD, P = 0.02) and MCP-1 (306 (204–362.5) pg/ml in short-term PD and 339.5 (304.8–644.8) pg/ml in long-term PD, *P* = 0.04) levels were significantly different between the short-term and long-term PD groups (Table 2). PD duration was positively correlated with plasma endotoxin (*r* = 0.479, *P* = 0.016) and MPC-1 (*r* = 0.486, *P* = 0.012) levels and was negatively correlated with age (*r* = −0.500, *P* = 0.009) and CCI (*r* = −0.389, *P* = 0.049) (Figure 1 and Table 3). PD duration was not correlated with serum iPTH, albumin, cholesterol, triglyceride, low-density lipoprotein cholesterol, high-density lipoprotein cholesterol, uric acid, normalized protein nitrogen appearance, total weekly creatinine clearance, or plasma IL-1β, IL-1RA, IL-6, IL-8, IL-10, IL-12p70, TNF, GM-CSF, or IFN-γ levels (Table 3, Figure S2 and S3).

**Table 3:** Correlations between PD duration, clinical parameters, and cytokine levels.

	Age	CCI	iPTH	Endotoxin	MCP-1
PD duration	*r* = −0.500 *P* = 0.009	*r* = −0.389 *P* = 0.049	*r* = 0.373 *P* = 0.061	*r* = 0.479 *P* = 0.016	*r* = 0.486 *P* = 0.012
Age	1	*r* = 0.339 *P* = 0.09	*r* = −0.266 *P* = 0.188	*r* = −0.313 *P* = 0.128	*r* = −0.100 *P* = 0.628
CCI		1	*r* = −0.357 *P* = 0.073	*r* = −0.235 *P* = 0.259	*r* = −0.091 *P* = 0.657
iPTH			1	*r* = 0.022	*r* = 0.069
				*P* = 0.919	*P* = 0.736
Endotoxin				1	*r* = 0.030
					*P* = 0.887

CCI, Charlson's comorbidity index; iPTH, intact parathyroid hormone; MCP-1 monocyte chemoattractant protein-1; PD, peritoneal dialysis; *r*, Spearman's rank correlation coefficient.

### Multivariate analyses of plasma endotoxin and MCP-1 levels

The multivariate linear regression models for endotoxin and MCP-1 levels are shown in Table 4. PD duration was independently associated with elevated plasma MCP-1 (beta coefficient  = 0.545, *P* = 0.039; Table 4).

**Table 4:** Multivariate linear regression analyses of factors associated with circulating endotoxin and MCP-1 levels.

	β coefficient	95% CI	P value
Dependent variable: MCP-1			
PD duration	0.545	0.17, 5.85	0.039
Dependent variable: Endotoxin			
PD duration	0.208	−0.18, 0.40	0.430

CI, confidence interval; MCP-1 monocyte chemoattractant protein-1; PD, peritoneal dialysis.

Age, gender, diabetes, Charlson's comorbidity index, serum albumin, high-sensitivity C-reactive protein; interleukin-1β, and interleukin-6 were included as variables in this model.

### Effects of short-dwell exchange on plasma endotoxin and MCP-1 levels

**Figure 2:**
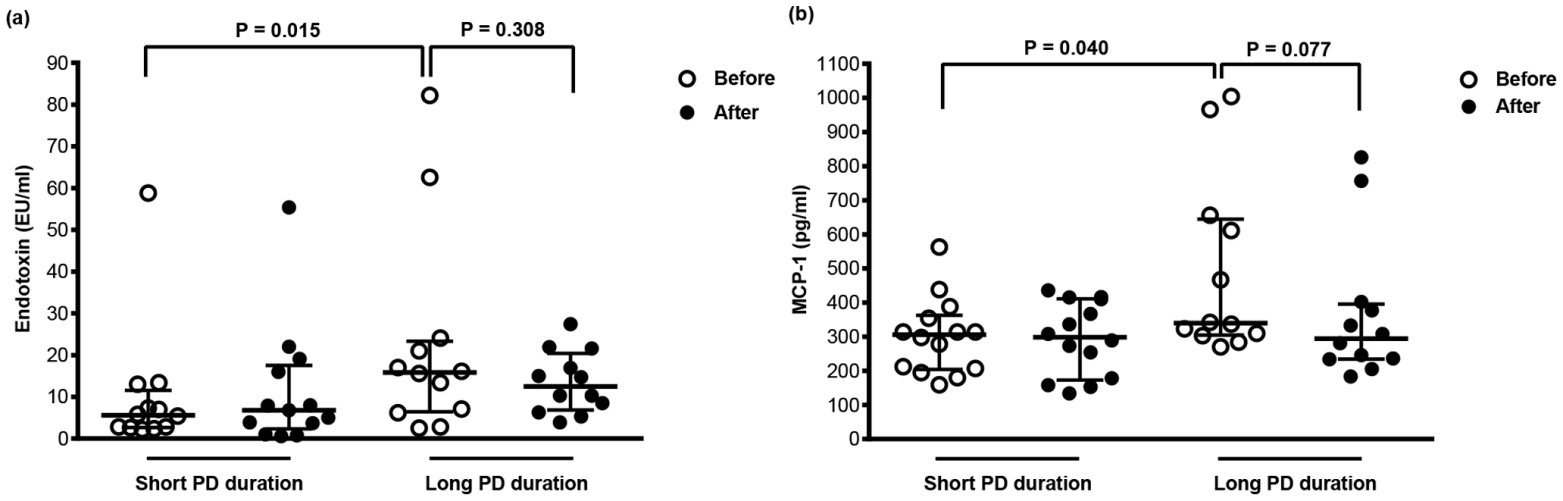
Comparison of circulating endotoxin (a) and MCP-1 (b) levels before and after a 4-h exchange between the short- and long-term PD groups. Circulating endotoxin and MCP-1 levels before a 4-hour exchange were significantly elevated in the long PD duration group. In the short PD duration group, plasma endotoxin and MCP-1 levels were not significantly different between before and after a 4-hour exchange. In the long PD duration group, plasma endotoxin levels were not significantly different after a 4-hour exchange (a). Plasma MCP-1 levels tended to decrease after a 4-hour exchange (b).

After a 4-h dwell, circulating endotoxin and cytokine levels were not significantly different between the short-term and long-term PD groups (Table 5). In the short-term PD group, plasma endotoxin and MCP-1 levels were not significantly different between before and after the 4-h exchange (Figure 2). In the long-term PD group, plasma endotoxin levels were not significantly different between before and after the exchange (Figure 2a). Plasma MCP-1 levels tended to decrease in the long-term PD group from 339.5 (304.8–644.8) pg/mL before the exchange to 294.5 (234.5–395.8) pg/mL after the exchange (13.3% reduction; P = 0.077; Figure 2b).

**Table 5:** Comparison of circulating endotoxin and cytokine levels after a 4-h exchange between the short- and long-term PD groups.

		PD duration			*P*-value
	Short (n = 14)	Baseline (% change)	Long (n = 12)	Baseline (% change)	
Endotoxin (EU/mL)	6.8 (2.4–17.6)	5.4 (2.5–10.2) (25.9%)	12.5 (6.9–20.4)	15.9 (6.4–23.3) (21.4%)	0.14
MCP-1 (pg/mL)	298.5 (173–411.3)	306 (204–362.5) (2.5%)	294.5 (234.5–395.8)	339.5 (304.8–644.8) (13.3%)	0.78
IL-1β (pg/mL)	5.7 (0–8.2)	4.8 (0–7.2) (18.8%)	3.1 (0.3–8.6)	2.7 (0.2–8.3) (14.8%)	0.86
IL-1RA (pg/mL)	149.6±18.5	129.3±14.3 (15.7%)	148±21.6	157.4±29.6 (6%)	0.64
IL-1β/IL-1RA ratio	0.027 (0–0.058)	0.027 (0–0.054) (0%)	0.028 (0.002–0.046)	0.012 (0.002–0.054) (133.3%)	0.94
IL-6 (pg/mL)	16.1 (10.1–21.6)	17.1 (9–21.5) (5.8%)	10.9 (5.4–16.4)	12.5 (6.9–16.5) (12.8%)	0.30
IL-8 (pg/mL)	22.1±2.3	21.6±1.8 (2.3%)	23.6±2.6	23.5±2.4 (0.4%)	1.00
IL-10 (pg/mL)	53.1 (22.6–93.9)	48.8 (21–69.9) (8.8%)	32.7 (13.6–85.7)	31.6 (18.1–73.3) (3.5%)	0.72
IL-12p70 (pg/mL)	18.8 (2–28.4)	16.8 (4.1–25.3) (11.9%)	10.2 (3.7–26.6)	10.6 (2.8–26.9) (3.8%)	0.86
TNF (pg/mL)	31.9±3.2	28.8±2.7 (10.8%)	32.7±3.3	30.6±3.0 (6.9%)	0.94
GM-CSF (pg/mL)	17.2 (0–29)	13.9 (3–24.6) (23.7%)	12.5 (1.2–29.8)	8.9 (0.8–26.4) (40.4%)	0.92
IFN-γ (pg/mL)	39.6 (4.7–68.6)	31.0 (10.4–54.2) (27.7%)	24.2 (9.9–63.2)	22.8 (9.9–62.9) (6.1%)	0.84

Values are median (interquartile range) or means±standard error of the mean.

% change, percentage change; GM-CSF, granulocyte-macrophage colony-stimulating factor; IFN-γ, interferon-γ; IL, interleukin; IL-1RA, interleukin-1 receptor antagonist; MCP-1, monocyte chemoattractant protein-1; TNF, tumor necrosis factor.

## Discussion

The current study revealed that patients receiving CAPD for >24 months had higher plasma endotoxin and MCP-1 levels than patients with a shorter PD duration. Circulating endotoxin and MCP-1 levels were also significantly correlated with the duration of PD. In addition, a short-dwell exchange was associated with a trend towards a decrease in plasma MCP-1 levels in patients on long-term PD.

Endotoxemia seems to be exaggerated by long-term PD and is associated with the peritoneal transport status. Elevated circulating endotoxin levels were also reported in patients with periodontal disease [27], diabetes mellitus [28], severe hepatic disease [29], or decompensated heart failure [30]. High endotoxin levels were also found in patients with advanced CKD and in patients on dialysis [10], [12]. Circulating endotoxin levels were almost 6 times higher in patients on dialysis compared with those who were not [10]. A population-based chronic disease risk survey demonstrated that high endotoxin level increased the risk for incident cardiovascular disease at 10 years (hazard ratio 1.82) [27]. Additionally, circulating endotoxemia was associated with systemic inflammation, malnutrition, atherosclerosis, and increased mortality in patients with advanced CKD and in patients on dialysis [10], [12]. Endotoxemia is thought to involve the translocation of endotoxin by a number of mechanisms. The authors [10] postulated that HD-induced splanchnic hypoperfusion may contribute to endotoxemia in HD patients while enteric venous congestion or edema may contribute to endotoxemia in PD patients. In addition, it is currently unknown which cytokine is involved or plays a dominant role in the etiology of systemic inflammation in patients on long-term PD. Our study revealed that circulating endotoxin and proinflammatory MCP-1 levels were elevated in patients on long-term PD. Conventional peritoneal dialysate fluids abolished in vivo leukocyte recruitment in response to lipopolysaccharide and may adversely affect the peritoneal host defense [31]. Additionally, patients who require PD for a long time may have severe periodontal disease [32], venous congestion or gastrointestinal edema because of reduced ultrafiltration, and the presence of more co-morbidities could exacerbate endotoxemia. LPS monomers are released from LPS aggregates by LPS-binding protein. These monomers are transferred to CD14 expressed on the membrane of circulating monocytes, resulting in monocyte activation and the production of proinflammatory mediators [33], [34]. The binding of LPS monomers to soluble CD14 expressed by endothelial cells induces cytokine production and atherosclerosis through the Toll-like receptor 4 signaling pathway [33], [35]. This signaling pathway induces MCP-1 production in murine peritoneal mesothelial cells and recruits leukocytes into the peritoneal cavity [36]. Higher endotoxin levels are associated with increased mortality risk in patients with CKD and dialysis patients [10]. Therefore, it seems feasible that patients on long-term PD may have more advanced atherosclerosis and cardiovascular disease, conferring increased risk of mortality.

Peritoneal fibrosis is a major cause of encapsulating peritoneal sclerosis, the most serious complications in patients on long-term PD, and is linked to ultrafiltration failure and higher mortality [37]. MCP-1 is closely related to inflammation and fibrosis induced by LPS, and is believed to be a good target for treating endotoxemia, sepsis, and peritoneal fibrosis [36], [38], [39]. Circulating MCP-1 level predicts LPS-induced murine systemic inflammation [40]. MCP-1 induces proliferation in human smooth muscle cells and is crucial to the initiation of atherosclerosis [41], [42]. Elevated circulating MCP-1 levels were associated with traditional risk factors for atherosclerosis and an increased risk of myocardial infarction or death in patients with acute coronary syndromes [43]. MCP-1 was also associated with an increased risk of recurrent cardiovascular events in diabetic patients with acute ischemic stroke [44]. In a previous study [45], plasma MCP-1 levels were 1.8-fold elevated in patients on dialysis compared with controls and the duration of dialysis was associated with elevated plasma MCP-1 levels and oxidative stress. Another study showed that MCP-1 concentrations in the PD effluent were positively related to serum MCP-1 and duration of PD [46]. Administration of LPS increases plasma MCP-1 levels *in vivo* [47], [48]. Endotoxin was also capable of inducing MCP-1 production in peritoneal mesothelial cells and may play an important role in peritoneal fibrosis according to prior *in vitro* studies [36], [49]. In the current study, we found that the PD duration was independently associated with plasma MCP-1 levels. Taken together, the higher circulating endotoxin and MCP-1 levels may reflect the inflammatory and fibrotic environment in the peritoneum, and might contribute to the poor outcomes in these patients.

Despite the increased levels of circulating endotoxin and MCP-1, we did not observe elevated levels of the anti-inflammatory mediators IL-10 and IL-1RA in patients on long-term PD. This imbalance between proinflammatory and anti-inflammatory cytokines had been reported in patients with chronic renal failure and dialysis patients [50], [51].

Previous studies have showed that epidermal growth factor, L-glutamine, oats supplementation, or zinc may reduce circulating endotoxin by preserving intestinal integrity in alcoholic liver disease [52]. Administration of rosiglitazone was also associated with a reduction in circulating endotoxin levels in patients with type 2 diabetes [28]. Additionally, sevelamer, a non-calcium-based phosphate binder, was reported to reduce plasma endotoxin levels in HD patients [13]. CAPD was thought to be associated with a slow but relatively constant clearance rate of large molecules [53]. MCP-1 has a much smaller molecular weight (13 kDa) than the LPS–LPS binding protein complex (70–80 kDa) or LPS aggregates (>1000 kDa) and is thought to be readily removed from the peritoneum by PD [54]–[56]. In the current study, we observed a trend towards a decrease in plasma MCP-1 levels (*P* = 0.077) but not in plasma endotoxin levels after a short-dwell exchange. Because of progressive ultrafiltration loss, gastrointestinal venous congestion or edema may be exaggerated during an overnight dwell with conventional glucose solutions in long-term PD patients. It is likely that endotoxin and MCP-1 are released during overnight dwells and MCP-1 may be removed by short-dwell exchanges in these patients. Automated peritoneal dialysis (APD) with short-dwell exchanges enhances ultrafiltration in many patients, especially in patients with high peritoneal solute transport [57]. Further studies are needed to clarify whether short-dwell exchange with APD reduces endotoxemia or MCP-1 and reduces the risk of atherosclerosis, peritoneal membrane failure, and mortality.

There are several limitations of our study. First, we only enrolled 26 patients and used a cross-sectional design rather than a longitudinal design. We did not perform a prospective follow-up and the small sample size reduced the statistical power of this study. Second, although the duration of PD was associated with circulating endotoxin and MCP-1 levels, we did not measure their levels in the PD effluent. Third, there may be some selection bias because patients on long-term PD were younger and their CCI was lower than patients on short-term PD. This selection bias may result in an underestimate of the true associations in both groups of PD patients. Fourth, single-point measurements of endotoxin and cytokine levels may result in the lack of a true ‘baseline’ or the possible inter-individual variability. Finally, we did not perform a pathological study of the peritoneum or determine the extent of peritoneal inflammation or fibrosis. Further studies such as multiple measurements of endotoxin and cytokine levels or obtaining serial blood/effluent samples during the short-dwell exchange would be needed to clarify these issues.

## Conclusions

This was the first study to show the effect of short-dwell exchange in terms of lowering circulating MCP-1 levels in patients on long-term PD. We found that the duration of PD was positively and significantly correlated with plasma endotoxin and proinflammatory MCP-1 levels, and that the duration of PD was independently associated with the MCP-1 level. Anti-inflammatory cytokine levels might not be significantly different between the short-term and long-term PD patients. Short-dwell exchange seemed to have favorable effects on circulating MCP-1 levels in patients on long-term PD. Further studies are warranted to elucidate whether short-dwell exchange reduces endotoxemia and MCP-1 and is a preferable regimen in patients on long-term PD.

## Supporting Information

Figure S1 **Principal-component analysis (PCA) loading plot (a) and scores plot (b).** (a) The first principal component (PC-1) accounts for 23.3% of the variance and is primarily defined by dialysate/plasma creatinine ratio at 4 hours (4-h D/P creatinine), body mass index (BMI), body surface area (BSA), Charlson's comorbidity index (CCI), glycated hemoglobin (HbA1c) and hemoglobin (Hb). The second principal component (PC-2) accounts for 16.9% of the variance and is defined by serum creatinine, phosphorus (P), age, weekly creatinine clearance (CrCL), uric acid, intact parathyroid hormone (iPTH), PD duration, ferritin, and albumin. The third principal component (PC-3) accounts for 11.2% of the variance and is defined by normalized protein nitrogen appearance (nPNA), serum calcium (Ca), cholesterol and white blood cell count (WBC). (b) Scores plot of 26 patients in a three-dimensional space derived from PCA. The scores plot shows grouping of the demographic and laboratory profiles between short (blue open circle) and long (green open triangle) PD duration groups.
(TIF)

Figure S2 **Correlations between the duration of PD and biological (a–f) and dialysis-related (g–i) parameters in patients on CAPD.** Duration of PD was not correlated with serum albumin (a), cholesterol (b), triglyceride (c), low-density lipoprotein cholesterol (d), high-density lipoprotein cholesterol (e), uric acid (f), normalized protein nitrogen appearance (g), or total weekly creatinine clearance (h).
(TIF)

Figure S3 **Correlations between the duration of PD and various serum cytokine levels except MCP-1 in patients on CAPD.** Duration of PD was not correlated with plasma interleukin-1β (a), interleukin-1 receptor antagonist (b), interleukin-6 (c), interleukin-8 (d), interleukin-10 (e), interleukin-12p70 (f), tumor necrosis factor (g), granulocyte-macrophage colony-stimulating factor (h), or interferon-γ (i) levels.
(TIF)
