# Supplementary figures and images for: Associations between the Duration of Dialysis, Endotoxemia, Monocyte Chemoattractant Protein-1, and the Effects of a Short-Dwell Exchange in Patients Requiring Continuous Ambulatory Peritoneal Dialysis
Source: PLoS One. 2014 Oct 6;9(10):e109558. doi: 10.1371/journal.pone.0109558 (PMC4186838; doi:10.1371/journal.pone.0109558)

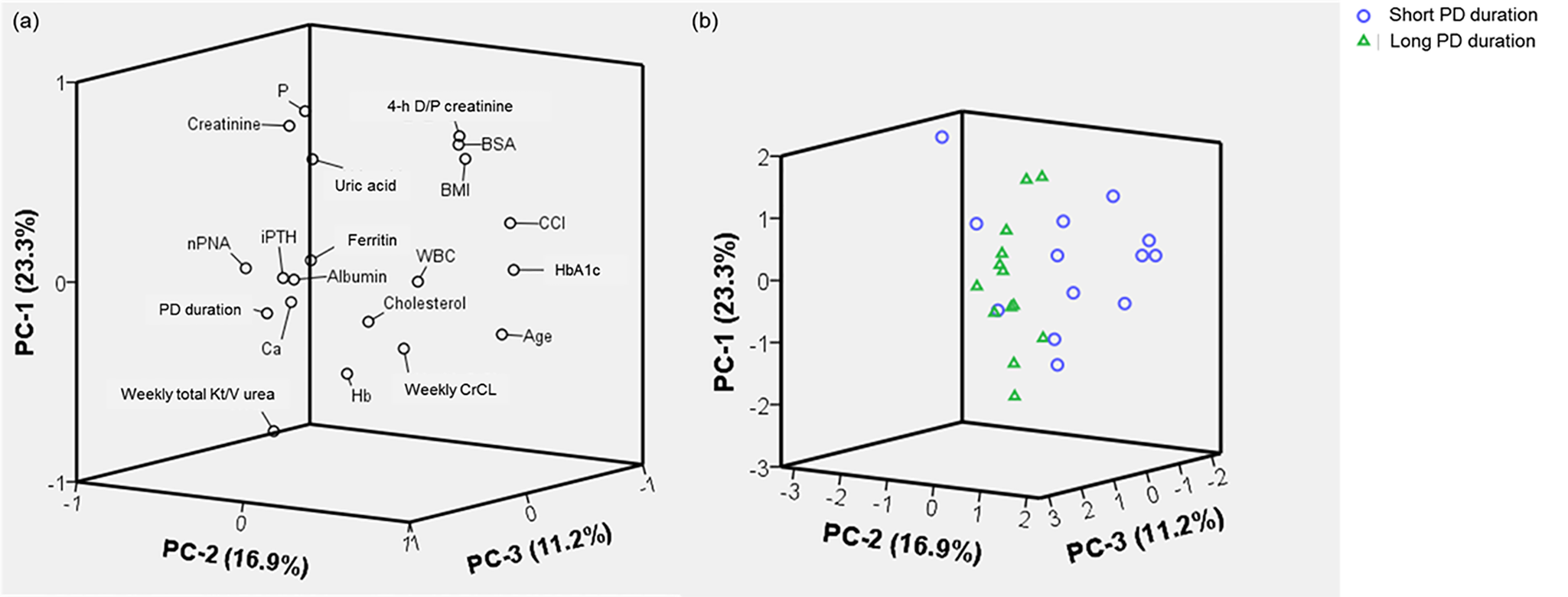

Supplement: Figure S1 — Principal-component analysis (PCA) loading plot (a) and scores plot (b). (a) The first principal component (PC-1) accounts for 23.3% of the variance and is primarily defined by dialysate/plasma creatinine ratio at 4 hours (4-h D/P creatinine), body mass index (BMI), body surface area (BSA), Charlson's comorbidity index (CCI), glycated hemoglobin (HbA1c) and hemoglobin (Hb). The second principal component (PC-2) accounts for 16.9% of the variance and is defined by serum creatinine, phosphorus (P), age, weekly creatinine clearance (CrCL), uric acid, intact parathyroid hormone (iPTH), PD duration, ferritin, and albumin. The third principal component (PC-3) accounts for 11.2% of the variance and is defined by normalized protein nitrogen appearance (nPNA), serum calcium (Ca), cholesterol and white blood cell count (WBC). (b) Scores plot of 26 patients in a three-dimensional space derived from PCA. The scores plot shows grouping of the demographic and laboratory profiles between short (blue open circle) and long (green open triangle) PD duration groups. (TIF) [file pone.0109558.s001.tif]

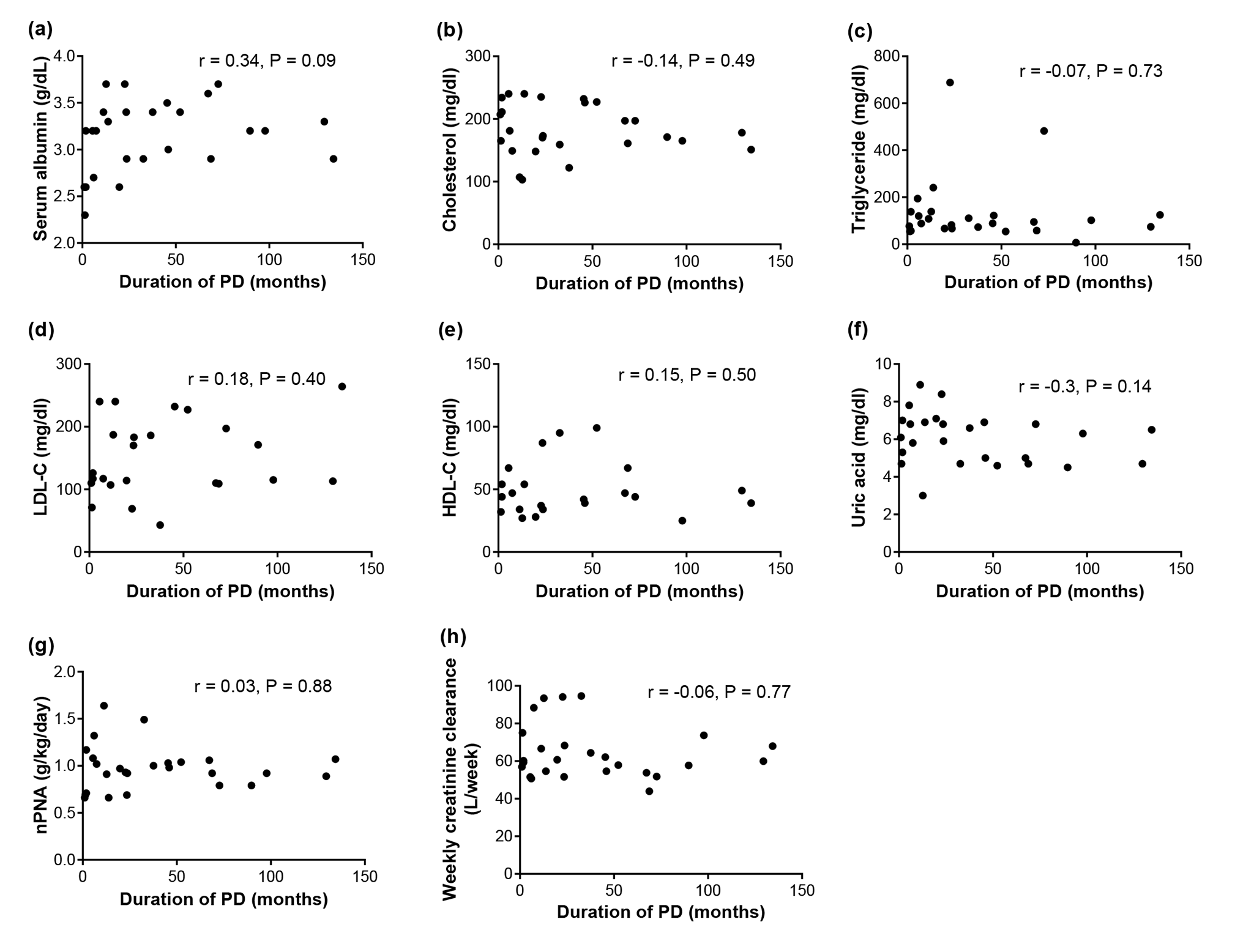

Supplement: Figure S2 — Correlations between the duration of PD and biological (a–f) and dialysis-related (g–i) parameters in patients on CAPD. Duration of PD was not correlated with serum albumin (a), cholesterol (b), triglyceride (c), low-density lipoprotein cholesterol (d), high-density lipoprotein cholesterol (e), uric acid (f), normalized protein nitrogen appearance (g), or total weekly creatinine clearance (h). (TIF) [file pone.0109558.s002.tif]

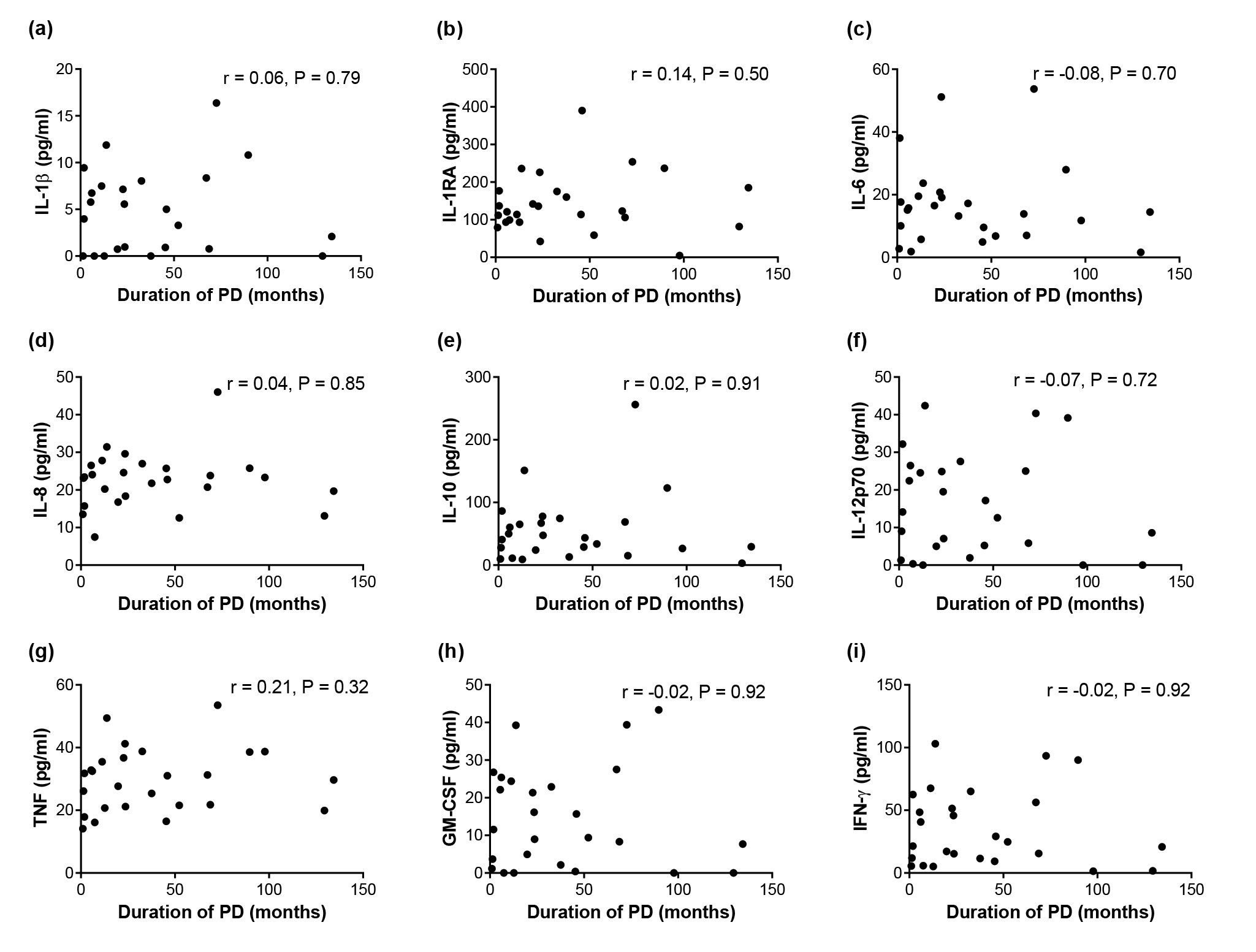

Supplement: Figure S3 — Correlations between the duration of PD and various serum cytokine levels except MCP-1 in patients on CAPD. Duration of PD was not correlated with plasma interleukin-1β (a), interleukin-1 receptor antagonist (b), interleukin-6 (c), interleukin-8 (d), interleukin-10 (e), interleukin-12p70 (f), tumor necrosis factor (g), granulocyte-macrophage colony-stimulating factor (h), or interferon-γ (i) levels. (TIF) [file pone.0109558.s003.tif]
